# A geospatial agent-based model of the spatial urban dynamics of immigrant population: A study of the island of Montreal, Canada

**DOI:** 10.1371/journal.pone.0219188

**Published:** 2019-07-24

**Authors:** Liliana Perez, Suzana Dragicevic, Jonathan Gaudreau

**Affiliations:** 1 Department of Geography, Laboratory of Environmental Geosimulation (LEDGE), Université de Montréal, Montreal (QC), Canada; 2 Department of Geography, Spatial Analysis and Modeling Lab, Faculty of Environment, Simon Fraser University, Burnaby (BC), Canada; University of South Carolina, UNITED STATES

## Abstract

Residential segregation into spatial neighborhoods and boroughs is a well-known spatial dynamic process that characterise complex urban environments. Existing models of segregation, including the pioneering Schelling ones, often do not consider all the factors that can contribute to this process. Segregation as well as aggregation emerges from local interactions among individuals, and is rooted in the complexity of social, economic and environmental interactions. The main objective of this study is to develop and implement a geospatial agent-based model to simulate the decision-making process of location of new household for incoming immigrant populations. Particularly this study aims to simulate and analyse the dynamics of the new immigrant populations arriving in the bilingual cities and boroughs of the island of Montreal. The model was implemented in NetLogo software, using real geospatial datasets. The obtained simulation results indicate realistic spatial patterns of spatial composition of the ethnographic fabric on the island of Montreal. The proposed model has the potential to be used as part of the city planning purposes.

## Introduction

International migration is a life-course strategy for many people all over the world. Common types of migration such as employment migration, marriage migration, family reunion, and retirement migration are indicators of how migration depends on events and stages in life. The life-course perspective on migration also points to the repetitive nature of migration [1,2]. One migration is often followed by another migration (e.g. return or onward migration) or even a sequence of migrations (e.g. circular migration) [3]. Whichever the reason, migratory phenomena have a great impact on the demographic, socio-territorial spatial and ethnographic configuration of cities [4]. The distribution of immigrant (an immigrant is defined as a person that moves to live permanently in a foreign country) population has been directly related to the level of development of the territory, in other words, there is a special interest on migratory flows and of course, spatially speaking, in places where these immigrants settled in relation to preferences, age, educational level, cultural level, etc. [5–7]. A nation built by immigrants where almost 22% of the total population recorded in 2016 correspond to immigrants [8,9], makes Canada the perfect place to study, analyse and model how people from different cultural and ethnolinguistic backgrounds can shape the spatial configuration of metropolitan cities. Of particular interest to this study, island of Montreal, Quebec, Canada, is recognized as a vibrant and cosmopolitan city, and by far the most bilingual city in Canada, where a majority of people speak both French and English, if not more languages [10]. For these reasons, the city attracts a vast majority of immigrants, from a diverse array of regions including Africa, Europe, Asia, Latin America and the Caribbean [11]. In Montreal, the immigrant population grew almost by 50% from 621,890 in 2001 to 936,305 in 2016, and it is recorded that every year it welcomes no less than 70% of new immigrants to Québec, making the city the second destination chosen by immigrant population in the country [11,12]. It is important to highlight that in 2017 the city of Montreal joined the Canadian cities of Toronto, Hamilton, London (Ontario) and Vancouver (British Columbia) in declaring itself a "sanctuary city" for unauthorized immigrants, ensuring that undocumented people can obtain social services without fear of deportation [13]. This latter initiative will likely attract more individuals without immigration status to participate in a new spatial and ethnographic configuration of the city.

Considering that immigrants accounted for almost 23% of Montreal’s population in 2011 [12] and 2016 [11], it seems appropriate to explore how the integration of newcomers affect or shape the spatial ethnographic fabric of the city. Settlement of immigrants in the city involves not only an autonomous or a forced decision, but also the evaluation of a series of principles, priorities and goals in life that each individual value differently. These individual desires can be reflected an organized or disorganized way, by making settlement decisions at very local, individual level, but creating an emerging spatial pattern of complex demographics at larger level of the urban space. This complex and multidimensional nature of the spatial dynamics involved in the decision-making process of where to locate when arriving into a foreign country can be analysed, represented and modeled using approaches such as geographic automata originated from the theory of complex systems [14].

The objective of this study is to use an agent-based modelling approach to develop and implement a spatially explicit model that allows the geospatial simulation of the decision-making process of newcomers arriving in the bilingual cities and boroughs of the island of Montreal and the resulting spatial segregation patterns. The model was implemented in NetLogo [15], a grid-based software, using geospatial raster datasets of 120m spatial resolution. The following sections give an overview of previous modeling approaches aiming to uncover the drivers of spatial segregation in urban environments. Next, the proposed and implement modeling approach to study the dynamics of immigrants in Montreal is presented together with the chosen approach to asses and interpret the results. Conclusions and future research avenues are then outlined.

## Literature review

Residential segregation is the grouping of individuals into well-defined spatial neighborhoods that emerges from local interactions amongst social, economic and environmental factors. Segregation as well as aggregation are now widely accepted as emergent patterns from complex urban systems and can be measured and modelled [16–18], indicating in some cases positive correlations between income level and urban sprawl and between income inequality and segregation [19,20]. Urban segregation is nowadays of particular interest and is becoming a key issue in global sustainable community planning and decision-making [17].

During the 1970s, Thomas Schelling developed the bounded-neighborhood and the spatial proximity models to represent residential segregation [21]. In the bounded-neighborhood model, the phenomenon of neighborhood tipping is explained. It was demonstrated how a neighborhood of one particular ethnicity could be drastically changed if others were to occupy it. A current example of this "tipping" could be 'white flight', where Caucasian residents leave an area after the arrival of African residents. With the spatial proximity model, Schelling uncovered a natural phenomenon in integrated communities. He found that residential segregation would still occur if the residents of one community tolerated to certain degree the presence of a different ethnicity but at the same time had a small preference for the same ethnicity. Together, these two models provide a more complete understanding of the patterns and persistence of segregation in communities [22].

The original Schelling model is based on spatial proximity and uses a checkerboard, and at that time was entirely against "letting a computer help with the work" [23]. It was reported that Schelling believed "programming could change one's view on the subject the program is about" and "programming forces programmers' view on the subject" and gives a "more general point of view". The Schelling models were ground-breaking, but they also had limitations. For example, the modeling approaches have not incorporated organized discriminatory action or income differences [24]. While the models do hold some degree of truth they are now considered outdated. Spielman and Harrison (2014) suggested that "the basic Schelling model is ill-suited to capture the complex residential landscape of the late 19th century" since "residential segregation is [also] a dynamic phenomenon" [25]. As a dynamic phenomenon, the segregation in a city may change drastically in a few decades [26]. Limitations of the models coupled with advances in spatio-temporal modeling have now allowed them to be updated and expanded.

The conceptual limitation is that the Schelling models focus primarily on segregation due to racial reasons and between only two ethnicities [27]. They do not include key driving factors such as religious, cultural and income differences, organized discriminatory actions, and social distance dynamics [24,28]. The role of immigrants also play a part in the segmentation process [26]. The current immigration rates in North America are much higher than forty years ago and there are currently much more "immigrant enclaves and ethnic communities" in North America [26]. This highlights the need for new models of segregation to account for ethnic diversity.

After executing their own analysis using a model similar to Schelling's, Spielman and Harrison (2014) found that, by "altering the built environment of a city and holding all social factors constant", they were able to produce results showing that the physical built environment of a city also plays a role in segregation. One of their examples involved the level of public transportation that was available. With greater public transit, those of the lower-income would have greater access to other housing markets. Logan et al. (2002) assumed that advances in public transit is one of the main reasons that ethnic communities have been able to spread out to encompass greater regions than in previous eras. Treating the agents in Schelling's model as particles, Vinkovic and Kirman (2006) [29]concluded that Schelling spatial proximity model is fairly similar to the Standard Model in the field of physics [30]. As the Standard Model has already been widely researched, this link could provide a good analytical framework for studying the basic Schelling Model and all of its variants.

Since the creation of the spatial proximity model, various researchers using diverse software tools have created several extended models. Gracia-Lázaro, Lafuerza, Floría, and Moreno, (2009) [31] incorporated the Axelrod model of cultural dissemination with the Schelling Model to include cultural-related factors into their model. Gilbert, (2002) [32] developed a model that showed the emergence of patterns and features. Several models were developed that focused on patterns of residential and ethnic dynamics [33–35]. These models incorporate additional factors and focused primarily on segregation. Kim, Tsou, and Feng (2015) [36] parallelize the Schelling Model, while Baldwin, Boardman, and Sauser (2013) [37] expanded the Schelling Model to a system of systems. These models were built with aim to increase the applicability of the Schelling Model in other fields. Zhang (2011), developed the unified model of all existing Schelling's Models to explain the causes of neighbourhood "tipping" and why the "tipping" process is difficult to reverse. While all of the other models are positive in the sense they provide further research and insight into their own respective categories, there is increased potential for progress if more researchers from varying fields are exposed to the Schelling Model. More links similar to the analogy that Vinkovic and Kirman (2006) had found between the basic Schelling Model and the Standard Model have the potential to be found. However, the issue related to cost and equipment remain a concern. As Kim et al. (2015) mentioned, to compute such a model would result in lengthy processing times; however, they have stated that increasing the number of CPU cores in the computer running the model would result in significantly shorter processing times.

In this regard, this research study aims to improve on existing models of segregation in three main ways. Firstly, an agent-based model is developed and implemented as a framework to capture the complexity of the segregation process. Secondly, multiple contemporary factors are considered in the formulation of the model to better describe the reality of immigrations settlement process. Thirdly, space and time are explicitly modeled with the use of real geospatial data and geographic information systems (GIS).

## Methodology

### Study area

**Fig 1:**
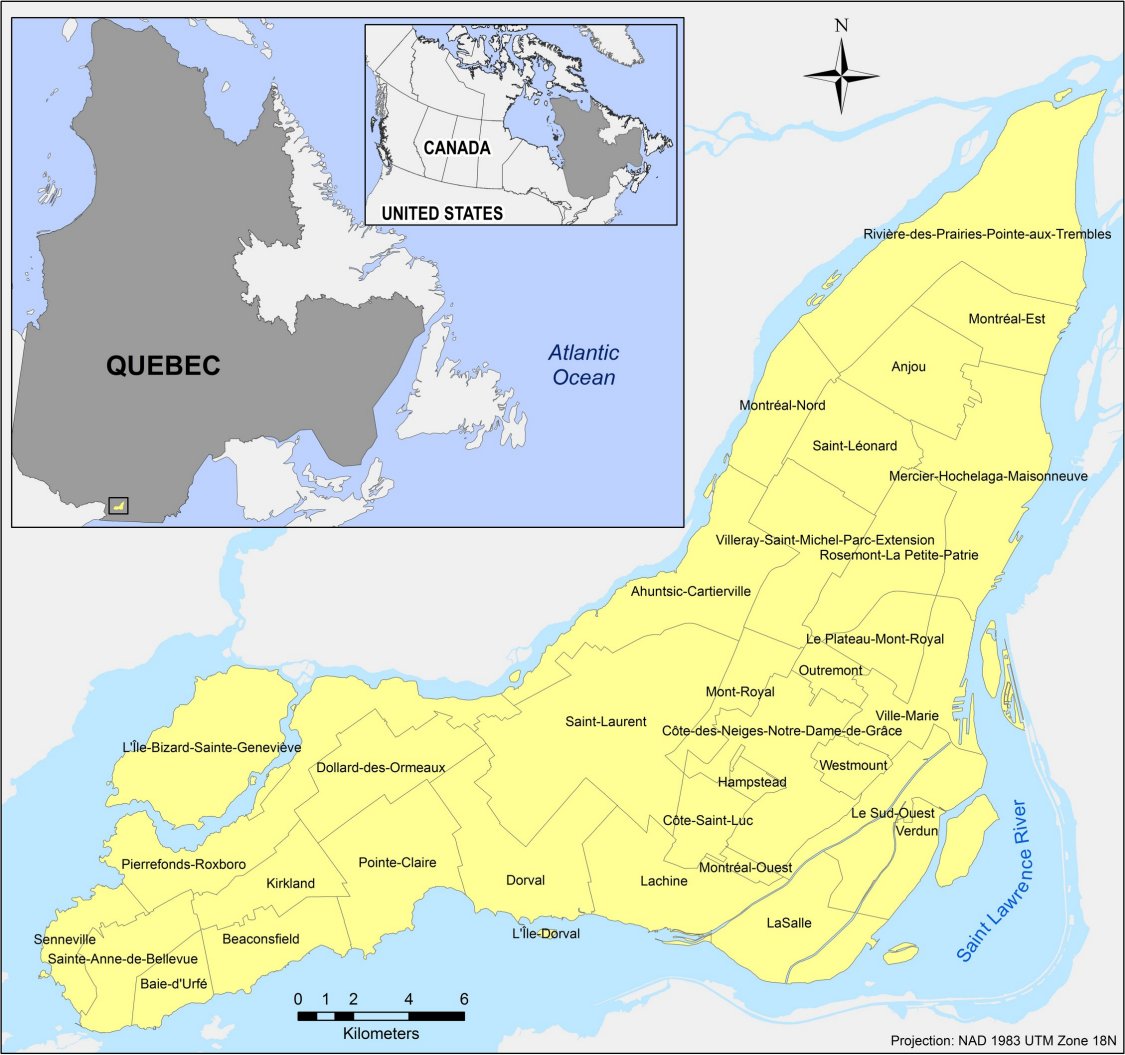
Study area—island of Montreal with composing cites and boroughs.

Situated on an island bordered by the St. Lawrence River, Montreal is the largest city in the French-speaking province of Quebec and the second largest in all of Canada with a population of around 4 million [11]. The city of Montreal (45.5017° N, 73.5673° W) is at once an island and an agglomeration that has been a preferred destination for immigrants over several decades already. As a city, it incorporates a territory spanned by the 19 boroughs (arrondissement in French) that make the fabric of Montreal (Fig 1). The Island of Montreal, meanwhile, includes the Ville de Montreal and 15 suburban cities; the territory as a whole form the agglomeration of Montreal. According to the most recent census 2016 [11], this territory is home to over twenty-three percent of individuals born abroad coming from diverse cultural backgrounds.

### Datasets

**Fig 2:**
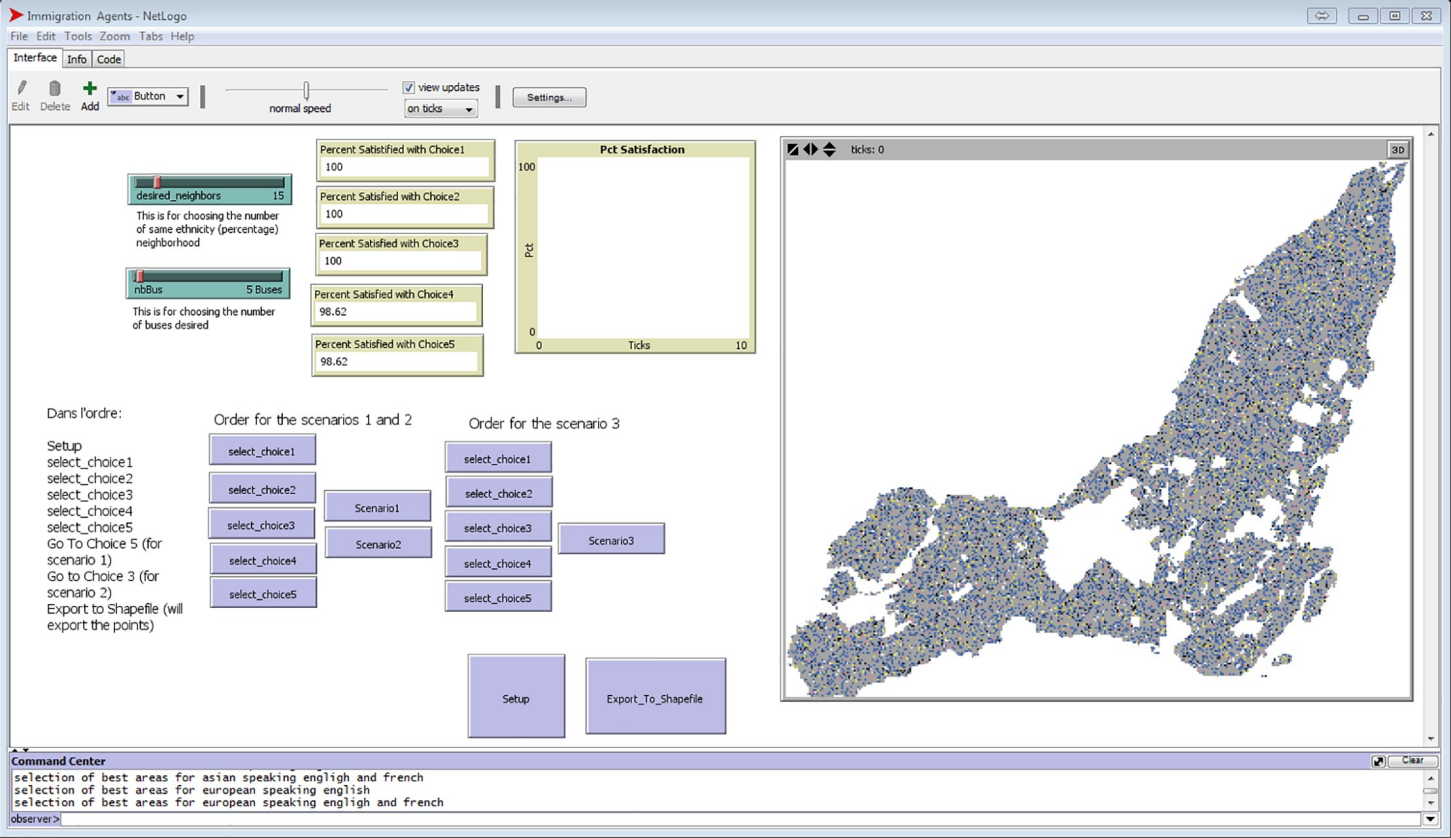
Simulation model graphical user interface (GUI) developed in NetLogo.

In order to simulate and analyse the spatial dynamics of immigrant population in the city of Montreal, census data, transportation network and land use data were used. Census data [8] were downloaded by dissemination area using the Canadian Census Analyser in spreadsheet format [38]. The variables extracted from the census and from the National Household Survey (NHS) [12] are presented in Table 1. The resulting database was joined to a vector GIS data layer with the different dissemination areas (DA) of Montreal, providing a vector-based thematic map with all the attributes by DA. Thematic maps by attribute were then converted to raster format with 120m spatial resolution min order to perform raster GIS analysis. Once the total population of each DA was available in raster, the variables were transformed to percentages of population per DA. Each converted variable was then used as model input. These transformations were necessary to implement the decision-making rules for the programmed immigrant agents. For example, if an immigrant agent prefers to live in an area where 30% of the neighborhood speaks the same language, he will evaluate the percent of speakers and not the raw number of speakers, since this number can vary between more populated and less populated DAs, knowing that they range from 400 to 700 people. In addition to census data, land use [39] and transportation network [40] data were also transformed into a raster format to be used as model input. While these layers could have been kept in a vector format, they were converted to raster where a value of 0 represents the absence and 1 represents the presence of a given variable. This process was necessary due to the grid-based configuration of the modelling software (NetLogo) used to implement the immigration model (Fig 2). Even though the Geographic Information Systems (GIS) extension in NetLogo [41] allows the ability to input vector-based GIS data layers, the initialization of the model proved to take a long computational time as NetLogo converts the vector files to raster GIS data format. Hence, in order to optimize the model’s performance, all GIS data layers were converted to raster before using them as model inputs.

**Table 1:** List of geospatial variables and attributes used for model implementation with sources of the datasets.

Variables	Attributes	Source
**Ethnicity (Country of origin)**	Africans Americans Asians Europeans	Census Canada, 2011, 2016
**Socioeconomic**	Median income	National Household Survey, 2011
**Spoken language**	English only French only English and French (both)	Census Canada 2011, 2016
**Demographic**	Total population Number of children Median age	Census Canada 2011, 2016
**Transportation Network**	Bus stops Subway stations	Société de Transport de Montréal, 2015
**Land-Use**	Commercial and/or employment area Parks Residential Public schools Private schools CEGEPS Universities	Ville de Montreal, 2015

### Agent-based model

The model proposed in this study was built to realistically represent the dwelling selection behaviour of visible immigrant communities arriving to a bilingual, multi-ethnic city, and the emerging patterns of spatial distribution or segregation in the city. Immigrants’ spatial decisions on the choice of the residential location are strongly influenced by the presence of family, friends and other people of the same ethnicity [42–45]. In addition to household characteristics, immigrants are usually motivated to immigrate and relocate to areas that will allow them to improve their wellbeing [46]. Access to good public schools, proximity to public transportation and green areas are other characteristics that will determine the residential choice of new immigrants. While some households may constraint their decision where to move because of limitations in income, other, less constrained households will try to select their location based merely on the economic status and overall income of their future neighbors. Considering all the factors playing in this process of decision, it is possible to develop a more sound understanding of how the spatial distribution and possible spatial segregation rises in a cosmopolitan city such as Montreal. These empirical observations are incorporated in the agent-based simulation model (ABM) [47]. Consisting of a population of individual actors or so called "agents", the island of Montreal as an environment, and a set of rules to allow the interaction between the agents and their environment, the actions of individuals in an ABM take place through the agents, which are simple, self-contained programs that collect information from their surroundings and use it to determine how to act.

Specifically, for the proposed model, immigrant individuals (also refer here as households) were considered as agents, while their decision process to select a place to live was simulated by a set of rules to follow in order to reach a degree of satisfaction with the chosen location. Attributes such as place of origin (continent), immigration category (as per categorisation by the CIC) [48], age, number of children, income, level of education and preferred spoken Canada’s official languages have been used to guide the decision making process of individuals. Table 2 provides details regarding agents’ attributes. This model is implemented using georeferenced GIS data layers of the island of Montreal, which acts as the environment where the “immigrant agents” interact and make decisions.

**Table 2:** Agents’ attributes and their different categories that represent the immigration profiles documented in the province of Quebec [49].

Agent Attributes	Categories	
Immigration category	Skilled worker Business Refugee	
Age	18–35 36–46	
Number of infants	0–3	
Income	Number of Family Members	Funds Required (in Canadian dollars)
	1	$11,931
	2	$14,853
	3	$18,260
	4	$22,170
	5	$25,145
	6	$28,359
	7 or more	$31,574
Education level	Superior No	
Language	French English Both	

The model is initialized as follows to represent Montreal’s immigration profile documented by the provincial government and based on the available census [49]. Groups of agents are classified by place of origin (Africa– 36.8%, Americas– 21.2%, Asia– 25.4%, and Europe– 16.6%). The initial number of agents that represent the city’s immigrant population is 12000. Once the model is initialized agents are randomly placed in the city based on the residential land use attribute of the spatial input raster and then the search for a house starts. Decision rules concerning the evaluation of distances use a dynamic circular neighbourhood, which size varies depending on the rule to be evaluated (e.g. distance to bus stop).

**Fig 3:**
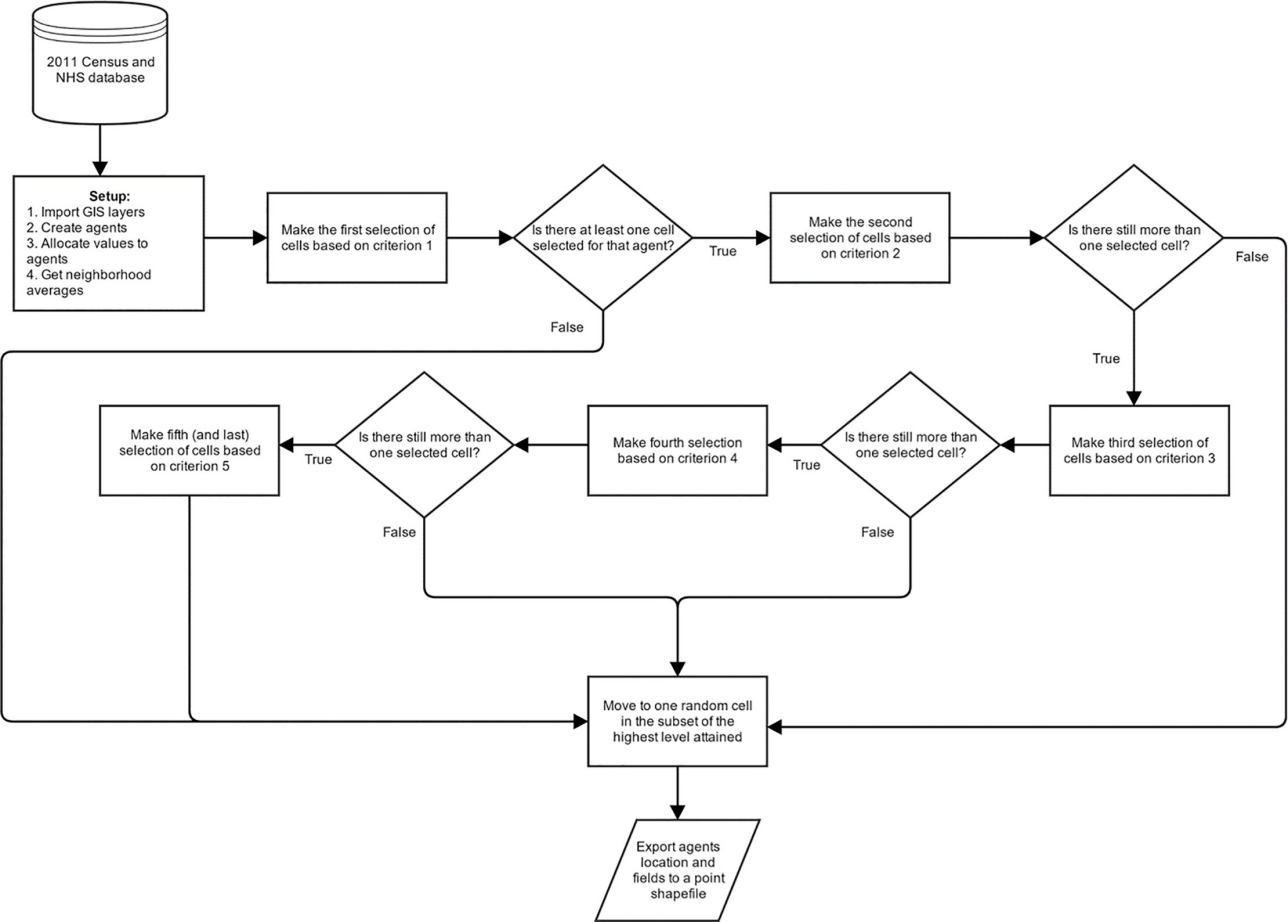
Geospatial agent-based model flow diagram that characterizes the decision-making process of immigrant agents to locate themselves within the city environment. The world “criterion” refers to the specific rule examined by the agent to make a decision.

The Fig 3 shows the flow diagram of rules implemented for agents’ behaviour that comprise the simulation model. At each time step, the immigrant agents check one-by-one all the decision rules that dictate their final choice of spatial location. Five rules are used to programme the immigrant agents to decide where to locate within the geographical space of the island of Montreal. These rules capture location choices. The decision rules are defined as follows:

1. **Immigrant agents will move to areas where the ethnolinguistic profile composition is tolerable**
  When an immigrant arrives to the city environment, the search for a new house begins. A location is considered favourable if at least a set percentage (%) of the surrounding (in a radius of 1 km) households are of the same ethnicity type as the immigrant agent searching for a place to move [5,50–55]. Likewise, the immigrant agent will prefer a location where more than 50% of the surrounding (in a radius of 3 km) households speak the same language spoken (not the mother tongue, but the language of preference to live in Canada) by the immigrant. Until these two conditions are not met, the agent will not evaluate the other parameters.
2. **The economic status of an immigrant agent will influence the location chosen**
  Immigrant agents with higher incomes are likely to locate themselves amongst households with high incomes [54,56–58]. Agents will select areas where households have similar incomes (between -10K and/or +5k of their income).
3. **Immigrant agents with infants will prefer areas where schools are accessible**
  If the immigrant agent has school-aged children, the proximity to schools is taken into consideration to decide the household spatial location. Desirable schools are generally thought to be within a 2km radius of the place selected [59–62].
4. **Neighbourhood quality will affect immigrant agents’ choice of location**
  Neighbourhood quality aspects such as access to restaurants, shops and green areas are amongst of the determining characteristics that play an important role in the selection of an neighbourhood to live [62,63]. A new location is thought to be favourable if there are green spaces within a radius 2 km and shops (commercial areas) within a radius of 4 km. This rule will only be evaluated by immigration agents with a business immigration category and for 50% of the immigrant with a skilled worker immigration category. Immigrant agents with a refugee category were not accounted into this rule due to their income constrains, based on the allowance given to government assisted refugees (GARs) [64,65].
5. **Immigrant agents will move to areas where there is access to bus and/or metro stations and bus stops**
  Normally, newcomers do not have the ability to posses the car, leaving with the only option of mobility by the use of public transportation [63,66,67]. Moreover, in cities like those on the island of Montreal, public transportation is preferred over a car due to difficulties associated with traffic congestions, parking and severe winter weather conditions. This rule ensures that a train station, metro station, and /or a bus stop is found within a 1km radius of the new location.

The evaluation of the rules (the number of rules and order depends on the scenario chosen from Table 3) is made per time step; once all the rules taken into consideration to move are evaluated, the immigrant agent will move. The total number of rules evaluated and their time steps represent a temporal resolution of one year; as shown in Fig 3, the model is initialized using census and NHS datasets of 2011, and four years are simulated to compare the simulation outputs with the census 2016 datasets. Temporal resolution was chosen considering the law from January 1^st^, 1974 which stipulates that population members of Quebec can move only on July 1^st^ each year [68], forcing everyone to move on the same day. Once the simulation begins and the agents move to the chosen location, they do not influence the geospatial variables and attributes (e.g. average income, total population, spoken language, etc.) of the geographic location they have selected. Table 3 provides information about three different scenarios and eight different tests designed to permit a better understanding of spatial distribution of immigrants when diverse priorities are considered in the decision-making process.

**Table 3:** Simulated scenarios and parameter variation tests.

Scenario	Description	
**Ethnolinguistic Scenario** **(Scenario 1)**	All the rules will be applied for the agent to make a decision (from one to five)	
**Parental Scenario** **(Scenario 2)**	Only rules 1, 2 and 3 will be important to make a decision	
**Parental/Economic Scenario** **(Scenario 3)**	All rules will be applied but the order will change to: Immigrant agents with infants will prefer areas where schools are accessible The economic status of an immigrant agent will influence the location chosen Immigrant agents will move to areas where there is access to bus metro stations and bus stops Immigrant agents will move to areas where the ethnolinguistic profile composition is tolerable Neighbourhood quality will affect immigrant agents choice of location	
**Test**	Parameters Values	
	Percentage of Neighbours	Number of Bus and/or metro stops
**Test1**	20	10
**Test2**	30	10
**Test3**	15	10
**Test4**	10	10
**Test5**	20	15
**Test6**	15	15
**Test7**	20	5
**Test8**	15	5

### Model evaluation

Despite evaluation steps such as calibration, sensitivity analysis and validation are key stages of agent-based modelling testing [69,70], models simulating human decision making could be somewhat challenging to evaluate. For this reason, the simulation outputs’ evaluation of the implemented model in this research study is accomplished by visually assessing the results from the scenarios and tests proposed in Table 3, and finding the ones that are similar to the actual spatial aggregation in the city. Likewise, the model is tested by assessing the level of diversity per dissemination area (DA), in terms of agents’ attributes, such as place of origin. Model outputs are then compared with the spatial diversity measure generated from census datasets (Canada Census 2016).

#### Spatial diversity assessment

To interpret the simulation outputs of the three different scenarios and tests proposed in Table 3, an index of diversity is developed. The diversity index is based on the Simpson’s Diversity Index [71], where each borough and city within the island of Montreal is ranked on the probability of randomly selecting two agents (representing immigrant individuals) and getting two agents from a different place of origin:

D=1−∑(nN)2 (1)

where *n* is number of agents of a given place of origin and *N* is the total number of agents from all origins, and *D* returns values between 0 and 1, indicating respectively from the least diverse (0) to the most diverse (1) areas on the island of Montreal.

## Results and discussion

**Fig 4:**
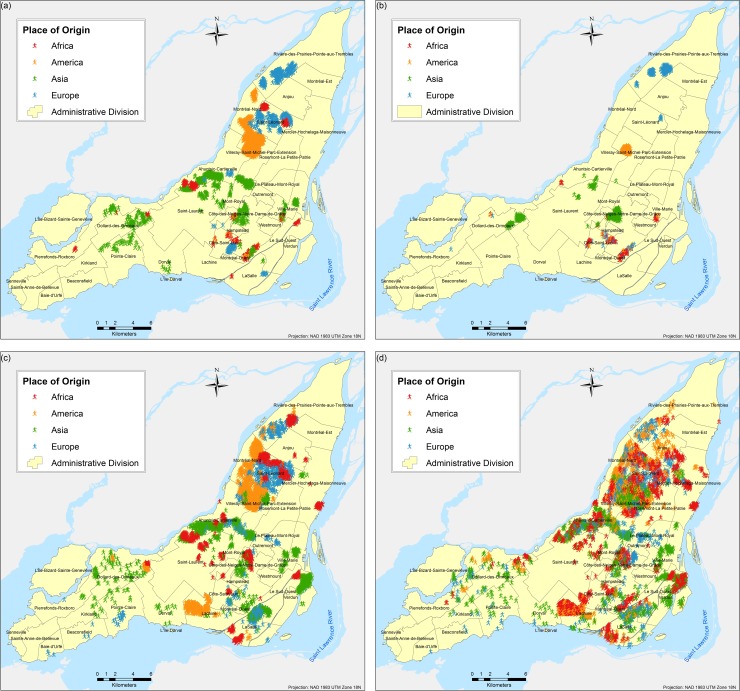
Simulated spatial distribution of immigrants after selecting their first place to live after arriving in the city. (a) Scenario 1 –Test 1, (b) Scenario 1 –Test 2, (c) Scenario 1 –Test 3, and (d) Scenario 1 –Test 4.

**Fig 5:**
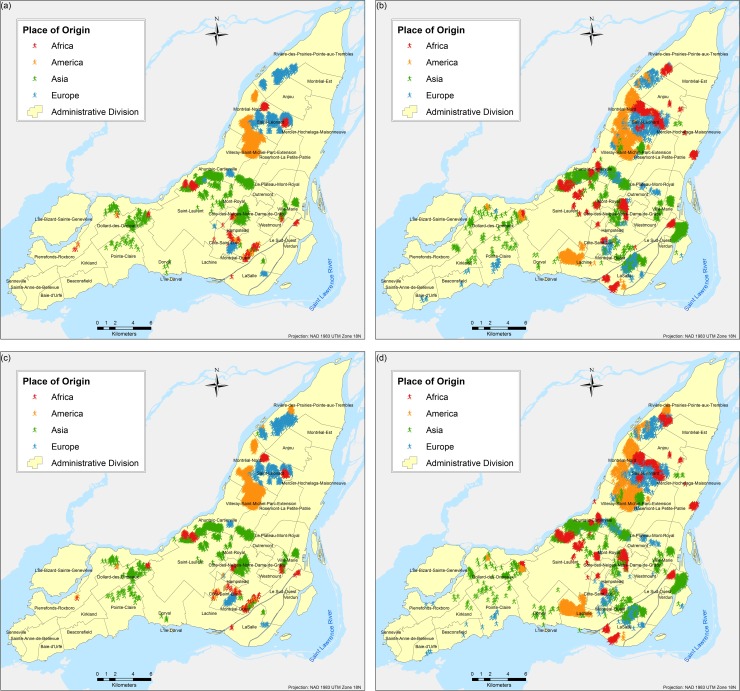
Simulated spatial distribution of immigrants after selecting their first place to live after arriving in the city. (a) Scenario 1 –Test 5, (b) Scenario 1 –Test 6, (c) Scenario 1 –Test 7, and (d) Scenario 1 –Test 8.

**Fig 6:**
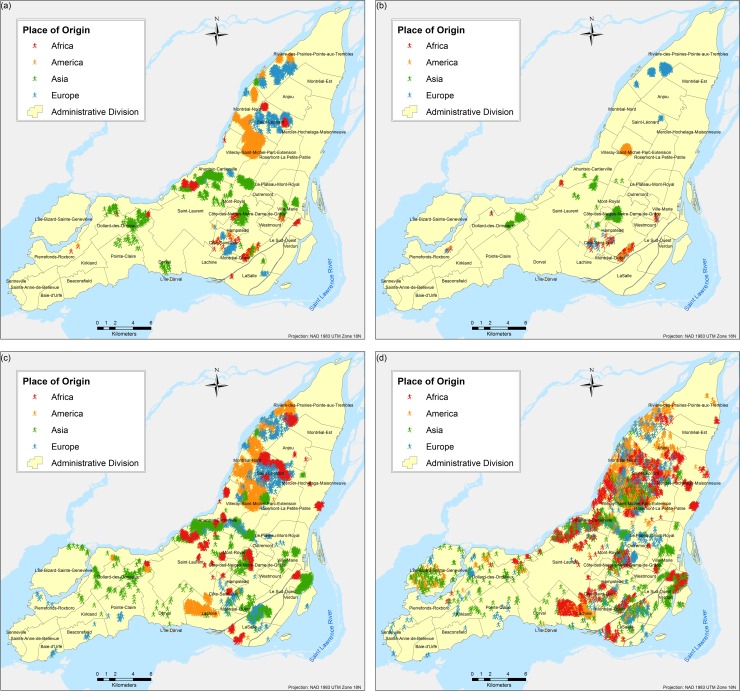
Simulated spatial distribution of immigrants after selecting their first place to live after arriving in the city. (a) Scenario 2 –Test 1, (b) Scenario 2 –Test 2, (c) Scenario 2 –Test 3, and (d) Scenario 2 –Test 4.

**Fig 7:**
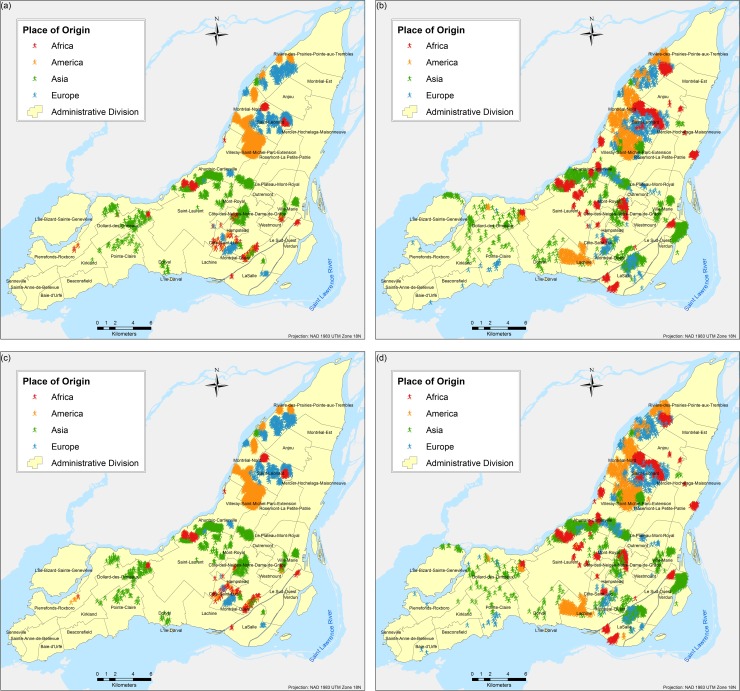
Simulated spatial distribution of immigrants after selecting their first place to live after arriving in the city. (a) Scenario 2 –Test 5, (b) Scenario 2 –Test 6, (c) Scenario 2 –Test 7, and (d) Scenario 2 –Test 8.

**Fig 8:**
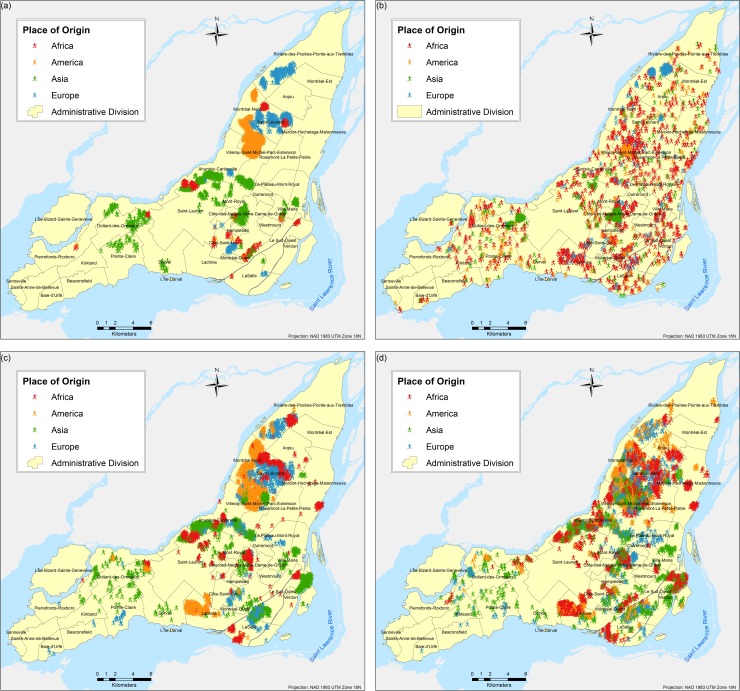
Simulated spatial distribution of immigrants after selecting their first place to live after arriving in the city. (a) Scenario 3 –Test 1, (b) Scenario 3 –Test 2, (c) Scenario 3 –Test 3, and (d) Scenario 3 –Test 4.

**Fig 9:**
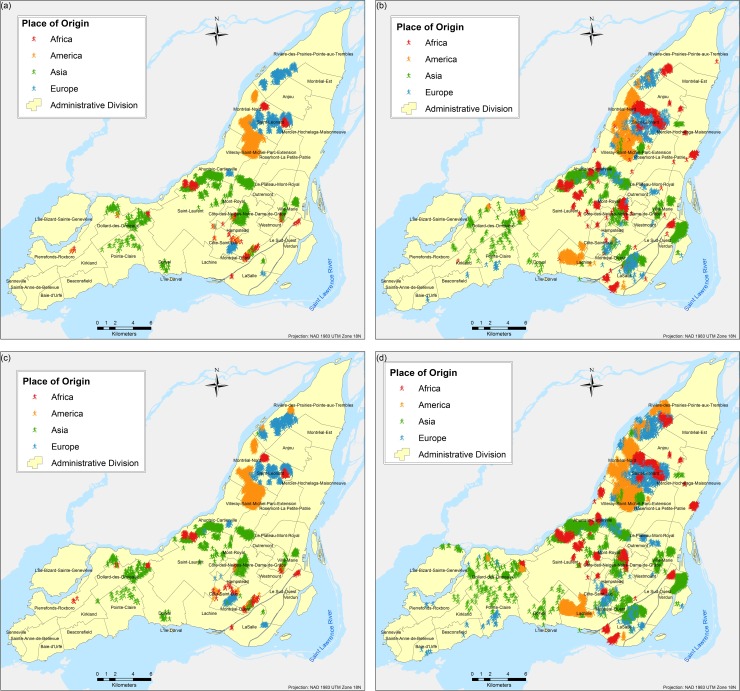
Simulated spatial distribution of immigrants after selecting their first place to live after arriving in the city. (a) Scenario 3 –Test 5, (b) Scenario 3 –Test 6, (c) Scenario 3 –Test 7, and (d) Scenario 3 –Test 8.

The results are obtained based on simulations for each of the three scenarios and eight different tests proposed. A total of twenty-four different combinations of simulations were performed (each corresponds to a different scenario experimented as per Table 3); due to the stochasticity of the model, each of the twenty four combinations were run fifty times respectively and the results presented here correspond to the weighted mean center of the fifty runs. Parameter variations are based on the following two parameters: (a) number of neighbours and (b) number of bus and/or metro stops, and they are used to calibrate the model and analyse the influence of them in the final spatial configure of the city. Figs 4–9 depict the simulation results for obtained values of different spatial distributions of immigrants based on the selection process as a result of their main preferences to settle in a new city. Visual inspection of the immigrant agents’ spatial distribution indicated that whenever agents are more flexible in terms of percentage of neighbours of their similar ethnolinguistic background the population distribution is less clustered and highly diverse per each spatial unit. Not surprising is the fact that among the several tests performed, Test 2 for the ethnolinguistic and parental scenario resulted in the most segregated and clustered pattern of immigrant agents’ distribution in the study area (Figs 4(B) and 6(B)). However, when the priorities in terms of decision rules change, like in the parental/economic scenario, the spatial distribution changes completely. Model outputs show that based on the decision from immigrant agents to prefer areas where schools are accessible (closer in distance), and then similar average income neighbours are favored, the spatial distribution of the immigrant population from an African and American origin is scattered throughout the city. Nevertheless, the spatial clustering and segregation pattern continues to be visible among the agents from an Asian and/or European origin under the simulation of Scenario 3 –Test 2 (Fig 8(B), see Table 3 for set of rules description).

**Fig 10:**
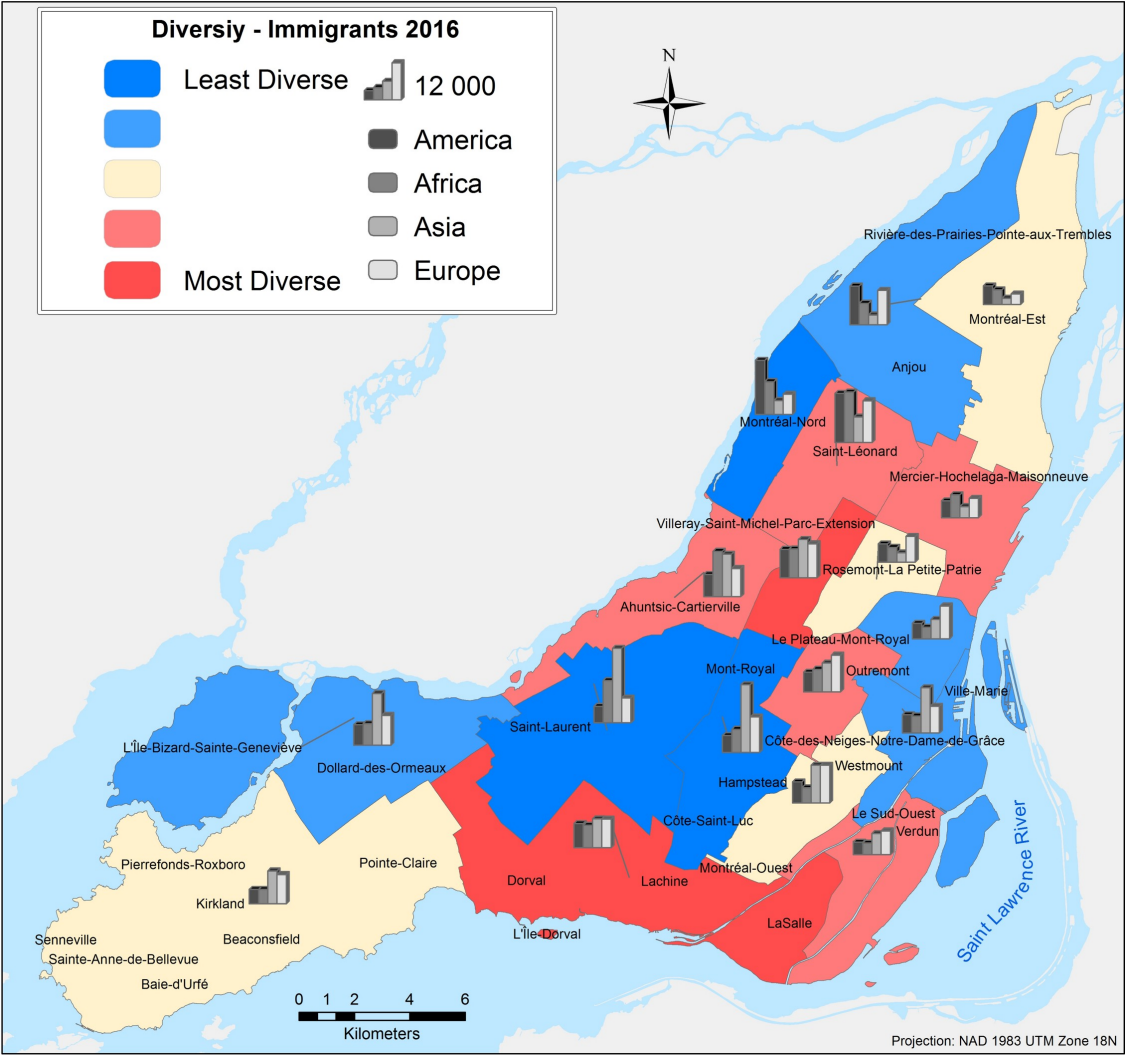
Spatial diversity map base on real data from Canada Census for the year 2016 [11].

An interesting outcome is observed in the simulation of Scenario 2 –Test 4 (Fig 6(D)), where importance is given to areas with better school accessibility. In that particular output, specifically for the geographic location of the borough L'Île-Bizard-Sainte-Geneviève, the immigrant agents’ distribution is concentrated within the island and corresponds to the real (census 2016) distribution of population, mostly from Asian origin, for both real (Fig 10) and simulated (Fig 6(D)) immigrant population distribution.

Overall, for the three simulated scenarios, the Test 4 (Figs 4(D), 6(D) and 8(D)) produces the least aggregated spatial distribution of immigrant agents, confirming that when agents are programmed with a higher level of tolerance in terms of neighbourhood diversity the emergence of spatial segregation is not evident.

**Fig 11:**
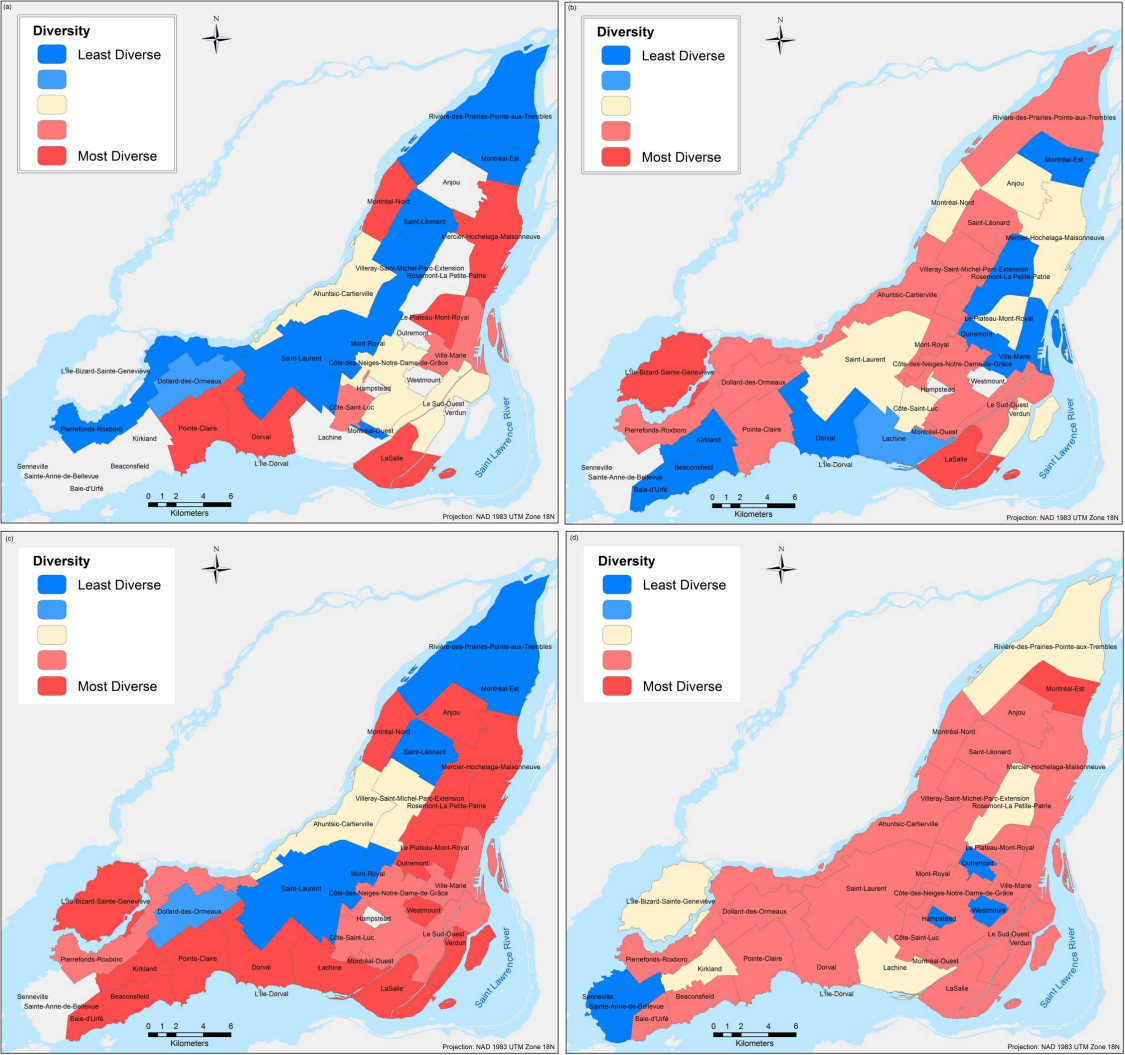
Spatial diversity mapping corresponding to the following combinations simulated: (a) Scenario 1 –Test 2, (b) Scenario 1 –Test 6, (c) Scenario 2 –Test 2, and (d) Scenario 3 –Test 2.

Validation results are obtained by computation of the proposed spatial diversity index using the real census data from 2016 and the simulated results for each of the twenty-four different combinations. Although the computation of the index was made for each DA, the results shown are aggregated by Federal Electoral Districts (FED) to allow easier visual interpretation of the simulated outputs. Fig 11, presents only four selected simulations that show similar spatial diversity patterns for different specific boroughs in the island of Montreal.

Although there is not one single scenario that can represent the spatial diversity of immigrants’ distribution in the Island of Montreal, there are key scenarios with specific parameter variations (represented by different tests) that resemble the real spatial segregation observed in the study area. Among the four combinations presented in Fig 11, the first one (a) corresponding to the Scenario 1 –Test 2, is the one that best represents the real spatial diversity captured by the census data. Specific boroughs on the island where the low spatial diversity is well captured are Dollar-des-Ormeaux, Saint-Laurent, Mont-Royal, and Rivière-des-Prairies-Pointe-aux-Trembles, while the high spatial diversity is well captured as well for the boroughs of Dorval, LaSalle, Le Plateau-Mont-Royal, and Mercier-Hochelaga-Maisonneuve. The Scenario 1 –Test 6 is presented in Fig 5(B), and it captures the high spatial diversity of boroughs such as Côte-des-Neiges-Notre-Dame-de-Grace (CD-NDG), well known in the island for is great diversity of habitants and mainly configured by immigrants’ communities from various origins. The values observed for the high diversity of other boroughs such as Ahuntsic-Cartierville, Le Sud-Ouest, Saint-Léonard, and Villeray-Saint-Michel-Parc-Extension are also well simulated for the latest combination discussed. The most important characteristic of Test 6 is the high percentage of tolerance that allows the immigrant agent to select a place to settle with a lower number of neighbours of similar characteristics to itself. Scenario 2 –Test 2, presented in Fig 11(C) overestimates diversity for the majority of the boroughs on the island, probably because only the first three rules are evaluated to make a decision to choose a place, not considering the quality of the chosen neighbourhood. Nevertheless, this combination produces the high spatial segregation found in the boroughs of Saint-Laurent and Mont-Royal.

Finally, the last combination presented in Fig 11(D) is generally very different from the Canada Census 2016 data. However, house market tendencies are emerging and matching the local and community knowledge that are present on the island of Montreal. Particularly, for three administrative areas of Outremont, Westmount and Hampstead the obtained simulation results as highly segregated and hence the calculated spatial diversity is very low, which is their well-known characteristic. For example, the Town of Hampstead is distinguished for having the highest percentage of Jewish residents of any city in Canada, and the third highest worldwide outside Israel, where seventy percent of the total population speaks only English [72]. Another important characteristic of this town is the average house prices, which are known to be among the highest on the island of Montreal. On the other hand, the residential borough of Outremont is largely inhabited by Francophone and where the house market prices for a duplex are around the million Canadian dollars. Lastly, the affluent city of Westmount, traditionally knowns as a wealthy and predominantly Anglophone area on the island of Montreal, registers one of the highest in North America with and income per-capita for a household of $210,120 [11].

## Conclusions

In this study, an agent-based model was developed and used as the framework to capture and analyse the complexity of the spatial segregation process in the context of the metropolitan city of Montreal. In order to do so, multiple contemporary variables are considered in the formulation of the model, and space and time are explicitly modeled in the process. Of particular interest to this research, is the simulation of the decision-making behaviour observed from new immigrants in a metropolitan setting. Model implementation was accomplished using NetLogo, a raster-based modelling software, which allows to program immigrant agents’ decision rules based on their knowledge about the characteristics of their future neighbours and neighbourhoods, as well as their tolerance in terms of their preferences to select a place to live. The model is initialized based on the migration profile reported by the provincial government in 2014, and it takes into account information such as continent of origin, immigration category, age, number of kids, average income, level of education and official spoken language. Considered the most bilingual city in Canada, Montreal offers a good simulation setting to test different scenarios of preference among new immigrants that differ from other cities in the Canadian migration context. Simulation results indicate that modelled dwelling selection among new comers in the island of Montreal; largely correspond to the real decisions observed in immigrants arriving to the island. Among the various tested scenarios, the ones that resulted in more aggregated spatial distributions, and hence less diverse, represent closely the real data collected by the Census 2016.

This research study brings two new contributions to the literature body of agent-based modeling, spatial segregation and immigration. First, the proposed model brings back the idea proposed by Schelling and expands it by explicitly modeling the geography of residential segregation among immigrants in a real context of a cosmopolitan, bilingual city. Not only is a real spatio-temporal framework implemented but also immigrant agents are programmed in a way that resemble different decision scenarios that are considered important to decide where to settle in an unfamiliar environment. Second, this model can be further developed into a decision platform that could assist in the planning of available social services for new comers. Some limitations of this model are with respect to model validation. The input datasets required to run the model are census data, however, although datasets from the 2011 and 2016 census are available, their geographic structure is not the same due to a redistribution of federal electoral districts in 2013 and changes in the census mapping units. Some other limitations or specificities to be considered are the decision-making assumptions implemented to drive the behaviour of the agents. Although the rules were built from examining numerous published literature, these preferences may vary between different cities, countries as well as ethnicities, and it is important to acknowledge them. Nevertheless, if adopted as a potential planning tool, this model could be advanced by using targeted interviews of new immigrants to evaluate their decision-making process and preferences and therefore calibrate the model with more realistic data.
